# Antibody-Mediated Targeting of the FGFR1c Isoform Increases Glucose Uptake in White and Brown Adipose Tissue in Male Mice

**DOI:** 10.1210/en.2017-00591

**Published:** 2017-08-16

**Authors:** Jo E. Lewis, Ricardo J. Samms, Scott Cooper, Jeni C. Luckett, Alan C. Perkins, James D. Dunbar, Dennis P. Smith, Paul J. Emmerson, Andrew C. Adams, Francis J. P. Ebling, Kostas Tsintzas

**Affiliations:** 1 School of Life Sciences, University of Nottingham, Queen’s Medical Centre, Nottingham NG7 2UH, United Kingdom; 2 Lilly Research Laboratories, Indianapolis, Indiana 46285; 3 School of Medicine, University of Nottingham, Queen’s Medical Centre, Nottingham NG7 2UH, United Kingdom

## Abstract

The increased prevalence of obesity and its cardiometabolic implications demonstrates the imperative to identify novel therapeutic targets able to effect meaningful metabolic changes in this population. Antibody-mediated targeting of fibroblast growth factor receptor 1c isoform (FGFR1c) has been shown to ameliorate hyperglycemia and protect from diet- and genetically-induced obesity in rodents and nonhuman primates. However, it is currently unknown which tissue(s) contribute to this glucose-lowering effect. Thus, to elucidate this effect, we treated euglycemic mice with H7, a monoclonal antibody that selectively targets FGFR1c, and used whole-body positron emission computed tomography with a glucose tracer (^18^F-fluorodeoxyglucose). Treatment with H7 increased basal glucose uptake in white adipose tissue (WAT), brown adipose tissue (BAT), the brain, and liver but reduced it in the quadriceps muscles. Consequentially, blood glucose was significantly reduced in response to treatment. Under insulin-stimulated conditions, the effects of H7 were maintained in WAT, BAT, liver, and muscle. Treatment with H7 decreased triglyceride (TG) content and increased adipose TG lipase content in white adipose tissue, while increasing activation of acetyl coenzyme A carboxylase, suggesting futile cycling of TGs, albeit favoring net hydrolysis. We demonstrated, *in vitro*, this is a direct effect of treatment in adipose tissue, as basal cellular respiration and glucose uptake were increased in response to treatment. Taken together, these data suggest that antibody-mediated targeting of FGFR1c exerts its powerful glucose-lowering efficacy primarily due to increased glucose uptake in adipose tissue.

## Teaser

Targeting the FGFR1c isoform reduces blood glucose via increased uptake in adipose tissue. This is a direct effect of treatment and appears to lead to a futile (re)cycling of triglyceride content.

The fibroblast growth factor (FGF) family is a diverse array of autocrine, paracrine, and endocrine factors reported to play key roles in the development, growth, and metabolic homeostasis (1). The activity of FGFs is dependent upon engagement of tyrosine kinase FGF receptors (FGFRs), with the FGFR1c isoform being proposed to be the primary FGFR accounting for the metabolic actions of the so-called endocrine family of FGFs (2, 3). In accordance with its metabolic mode of action, the FGFR1c is expressed by several metabolically active organs, including white adipose tissue (WAT) and brown adipose tissue (BAT), as well as the hypothalamus (4), an area of the central nervous system associated with regulation of appetite. Recently, with the aim of understanding the role of the FGFR1c in the regulation of energy homeostasis, we used peripheral and central administration of a monoclonal antibody (H7) that selectively targets this isoform. We found that targeting of FGFR1c in the Siberian hamster, a natural model of adiposity, results in a profound antiobesogenic phenotype, accompanied by a substantial reduction in fed blood glucose levels and a clear suppression of daily food intake (5). In support of these data, administration of H7 to mouse models of obesity and diabetes reduces body weight, fat mass, blood glucose levels, and insulin resistance (6–8). However, it is unknown which tissue(s) are responsible for this antidiabetic activity. Mice with adipose-selective FGFR1 knockout are refractory to the antidiabetic activity of FGF21, a natural ligand for FGFR1c (8). Therefore, it appears that adipose tissue might be an important target for H7. Thus, the aim of this study was to ascertain the tissue-specific glucose uptake effects in response to peripherally targeting FGFR1c under basal and insulin-stimulated conditions, with particular emphasis on WAT and BAT utilizing *in vivo* and *in vitro* approaches. To account for any adverse effects of obesity-induced insulin resistance on tissue glucose uptake, a lean euglycemic animal model was selected to fulfill the objective of this study.

## Research Design and Methods

### Animals and experimental protocol

All studies were approved by the University of Nottingham Animal Welfare and Ethical Review Board and were carried out in accordance with the UK Animals (Scientific Procedures) Act of 1986 (project license PPL 40/3604). Age-matched adult male C57BL/6 euglycemic mice were randomly divided into one of two treatment groups in which they received a single subcutaneous injection of either vehicle (n = 8) or H7 (n = 8; 3 mg/kg), as previously described (6). After 24 hours, a subset of animals from each treatment group (n = 4 per group) were transferred to metabolic cages (Comprehensive Laboratory Animal Monitoring System; Linton Instrumentation, Linton, United Kingdom; Columbus Instruments, Columbus, OH) to measure daily energy expenditure. Seventy-two hours post injection, following a 4-hour fast, all animals were transferred to the nano–positron emission tomography/computed tomography (PET/CT) imaging unit (Mediso Medical Imaging Systems, Budapest, Hungary) to quantify glucose uptake in response to intraperitoneal infusion of ^18^F-fluorodeoxyglucose (^18^F-FDG) (10 MBq per animal) under basal (n = 4 per treatment) and insulin-stimulated conditions (subcutaneous injection, 0.75 U/kg, 10 minutes post ^18^F-FDG infusion, n = 4 per treatment). All scans lasted 60 minutes and were initiated 10 minutes post infusion of ^18^F-FDG. The magnitude of tissue-specific ^18^F-FDG uptake was expressed as the standard uptake value defined as the average ^18^F-FDG activity in each region of interest (in kBq/cm^3^) divided by the injected dose (kBq) and multiplied by the body weight of each animal (kg). Animals were subsequently euthanized, and blood was obtained, via cardiac puncture, to measure glucose (HemoCUE 201 System; HemoCUE, Angelholm, Sweden), plasma insulin, and tumor necrosis factor *α* (TNF-*α*) (Millipore, Billerica, MA). Triglyceride (TG) content of excised liver, adipose tissue, and muscle tissue was performed as previously described (9).

### Mitochondrial function assay

Mouse 3T3-L1 fibroblasts (ATCC, Manassas, VA) were differentiated for 10 days and treated with vehicle, 10 nM immunoglobulin G1 (IgG1), or 10 nM H7 for 24 hours, followed by assessment of glucose uptake as previously described (10) and mitochondrial function via the mitochondrial stress test (Seahorse XF Cell Mito Stress Test Kit; part no. 103015-100; Agilent Technologies, Inc., Wilmington, DE) (n = 14 per treatment).

### Western blotting

Proteins were extracted from WAT using a HEPES lysis buffer and quantified using the bicinchoninic acid protein assay (catalog no. 23227; Pierce, Thermo Fisher Scientific Inc., Waltham, MA). Western blotting was performed and resulting membranes were probed overnight at 4°C with primary antibodies (1:1000 dilution) to adipose TG lipase (ATGL; catalog no. ab109251; Abcam, Cambridge, UK), phospho-acetyl coenzyme A carboxylase (pACC; catalog no. 3661; New England Biolabs, Ipswich, MA), and cyclophilin B (CYC; 1:4000; catalog no. ab74173; Abcam). Blots were then washed and incubated for 1 hour with anti-rabbit (ATGL and pACC) and anti-mouse (CYC) horseradish peroxidase secondary antibody (Dako Denmark A/S, Glostrup, Denmark) at 1:2000 dilution. All immunoreactive proteins were detected using ECL Prime Western Blotting Detection Reagent (catalog no. RPN2232; GE Healthcare, Chicago, IL), quantified by densitometry using an Aida Image Analyzer (version 4.27), and normalized to CYC.

### Statistical analysis

Descriptive statistics (mean ± standard error of the mean) were generated using GraphPad Prism (Prism 6.0; GraphPad, San Diego, CA). Body weight, food intake, and metabolic cage data were analyzed using two-way (treatment × time) repeated measures analysis of variance (ANOVA). Tissue weights and protein content, blood glucose, plasma insulin and TNF-*α*, tissue TG content, and ^18^F-FDG uptake were analyzed using a Student *t* test. All image analysis was performed blinded to treatment using VivoQUANT software (inviCRO, Boston, MA). Glucose uptake in adipocytes was analyzed using one-way ANOVA. Maximal cell oxygen consumption rate (OCR), analyzed using both the area under the curve and the peak values (oxidative capacity), basal cellular respiration, adenosine triphosphate (ATP) generation, and nonmitochondrial respiration were analyzed using a one-way ANOVA. Statistical significance was declared at *P* < 0.05.

## Results

Body weight was decreased by approximately 14% compared with vehicle-treated controls (*P* < 0.0001). Similarly, food intake was reduced by 34% during the 72-hour experimental period (*P* < 0.0001), resulting in reduced visceral WAT weight (43%, *P* < 0.0001). Although there was a clear diurnal variation in whole-body energy expenditure in both vehicle- and H7-treated mice, there was no effect of acute treatment with H7 on energy expenditure during the final 24 hours of the experimental period.

**Figure 1.**
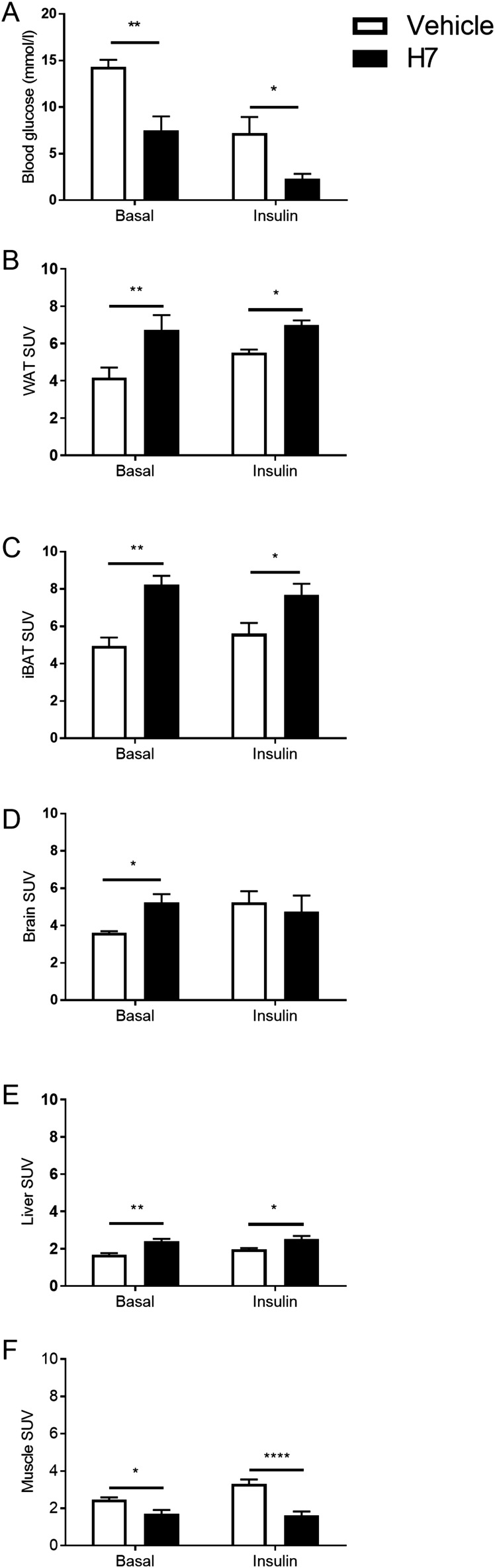
Effect of systemic treatment with a monoclonal antibody that targets the FGFR1c (H7) on blood glucose and basal and insulin-stimulated glucose uptake [expressed as standard uptake value (SUV)] in WAT, interscapular BAT (iBAT), brain, liver, and skeletal muscle in mice. (A) Blood glucose of adult male mice treated with vehicle or H7. Values are group mean ± standard error of the mean (SEM) (n = 4 per treatment). *P < 0.05, **P < 0.01 vs vehicle. (B–F) ^18^F-FDG uptake in (B) WAT, (C) iBAT, (D) brain, (E) liver, (F) and quadriceps muscles treated with vehicle or H7. Values are group mean ± SEM (n = 4 per treatment). *P < 0.05, **P < 0.01, ****P < 0.0001 vs vehicle.

Treatment with H7 resulted in a 70% decrease in plasma insulin and a 50% decrease in TNF-*α* (*P* < 0.05, respectively). Similarly, basal blood glucose concentrations were also reduced by 53% in animals treated with H7 (Fig. 1A; *P* < 0.01), and they were further reduced (by 63%) in the insulin-stimulated group (Fig. 1A; *P* < 0.05).

**Figure 2.**
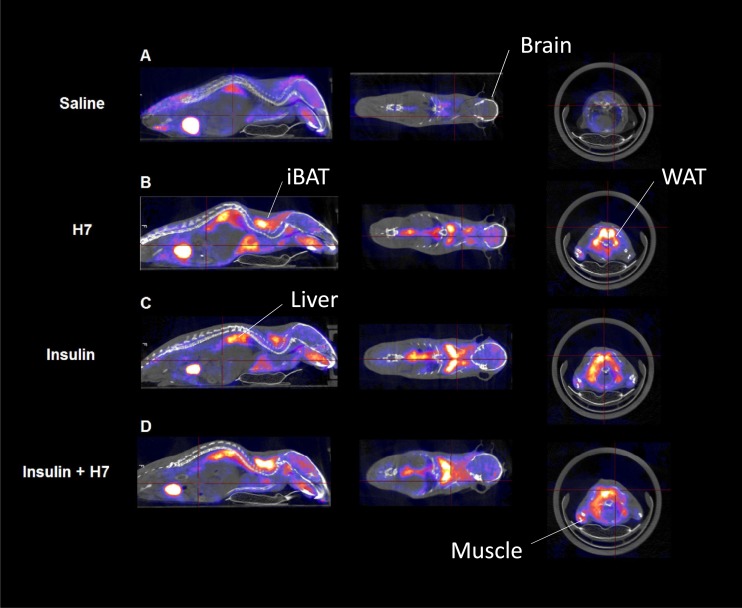
Representative whole-body PET/CT images of euglycemic mice receiving ^18^F-FDG treated with (A) vehicle (saline), (B) H7 + vehicle (saline), (C) vehicle (saline) + insulin, and (D) H7 + insulin. iBAT, interscapular BAT.

In WAT and interscapular BAT, treatment increased ^18^F-FDG uptake compared with vehicle-treated controls (Fig. 1B and 1C; *P* < 0.01). This effect was maintained under insulin-stimulated conditions (Fig. 1B and 1C, *P* < 0.05). In the brain, under basal conditions, treatment with H7 significantly increased ^18^F-FDG uptake compared with vehicle-treated controls (Fig. 1D; *P* < 0.05). However, there was no difference under insulin-stimulated conditions (Fig. 1D). Similarly, treatment with H7 increased hepatic ^18^F-FDG uptake compared with vehicle-treated controls both under basal (Fig. 1E; *P* < 0.01) and insulin-stimulated conditions (*P* < 0.05). However, in the muscle, treatment with H7 reduced ^18^F-FDG uptake compared with vehicle-treated controls in both basal (Fig. 1F; *P* < 0.05) and insulin-stimulated conditions (Fig. 1F; *P* < 0.0001). Representative images are shown in Fig. 2.

**Figure 3.**
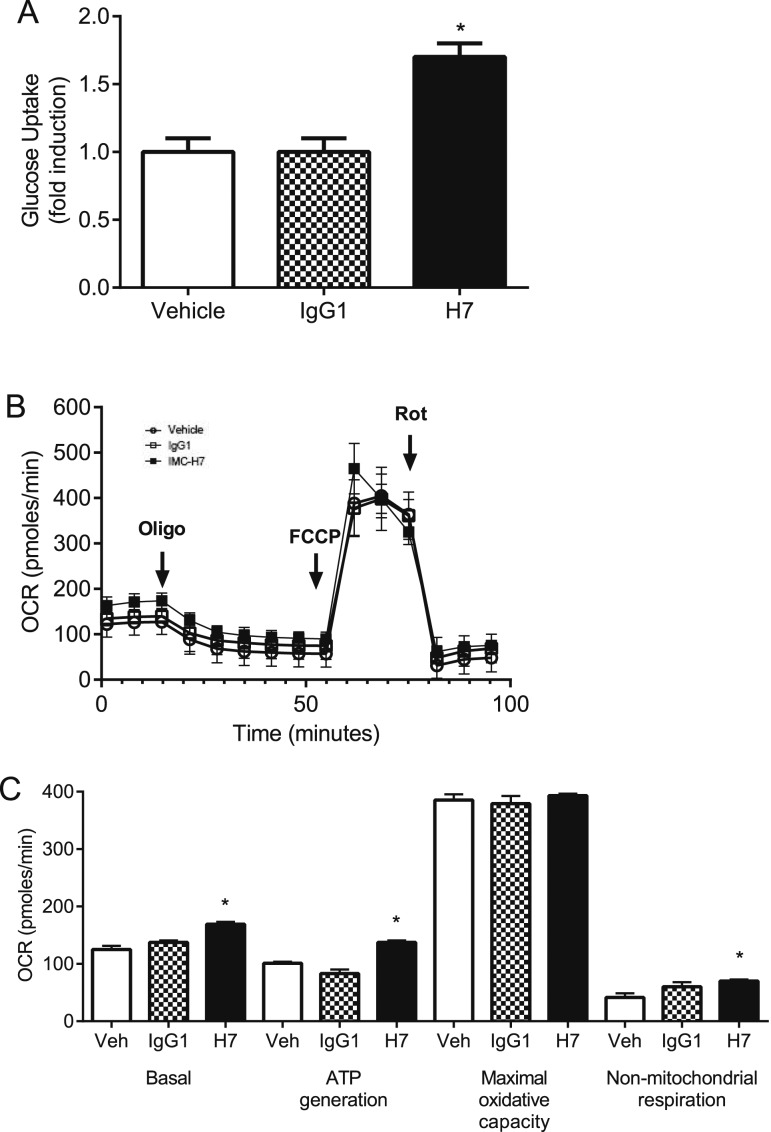
*In vitro* treatment of adipocytes with a monoclonal antibody that targets the FGFR1c (H7) increases glucose uptake and basal respiration but not maximal OCR. (A) Glucose uptake in adipocytes treated with vehicle, IgG1, and H7. Values are group mean ± standard error of the mean (SEM) (n = 14 per treatment). *P < 0.05 vs vehicle/IgG1. (B) OCR in adipocytes treated with vehicle, IgG1, and H7. Values are group mean ± SEM (n = 14 per treatment). (C) Basal cellular respiration, ATP generation, maximal oxidative capacity, and nonmitochondrial respiration in adipocytes treated with vehicle, IgG1, and H7. Values are group mean ± SEM (n = 14 per treatment). *P < 0.05 vs vehicle and IgG1. FCCP, carbonyl cyanide-4 (trifluoromethoxy) phenylhydrazone; Oligo, oligomycin; Rot, rotenone plus antimycin; Veh, vehicle.

In line with *in vivo* data, treatment with H7 significantly increased glucose uptake into 3T3-L1 adipocytes when compared with vehicle and IgG1 (Fig. 3A; *F* = 16.33, *P* < 0.05), whereas there was no effect of treatment on maximal adipocyte OCR (oxidative capacity) (Fig. 3B and 3C). However, H7 increased (*P* < 0.05) basal cellular respiration, ATP generation, and nonmitochondrial respiration when compared with vehicle and IgG1 (Fig. 3C).

**Figure 4.**
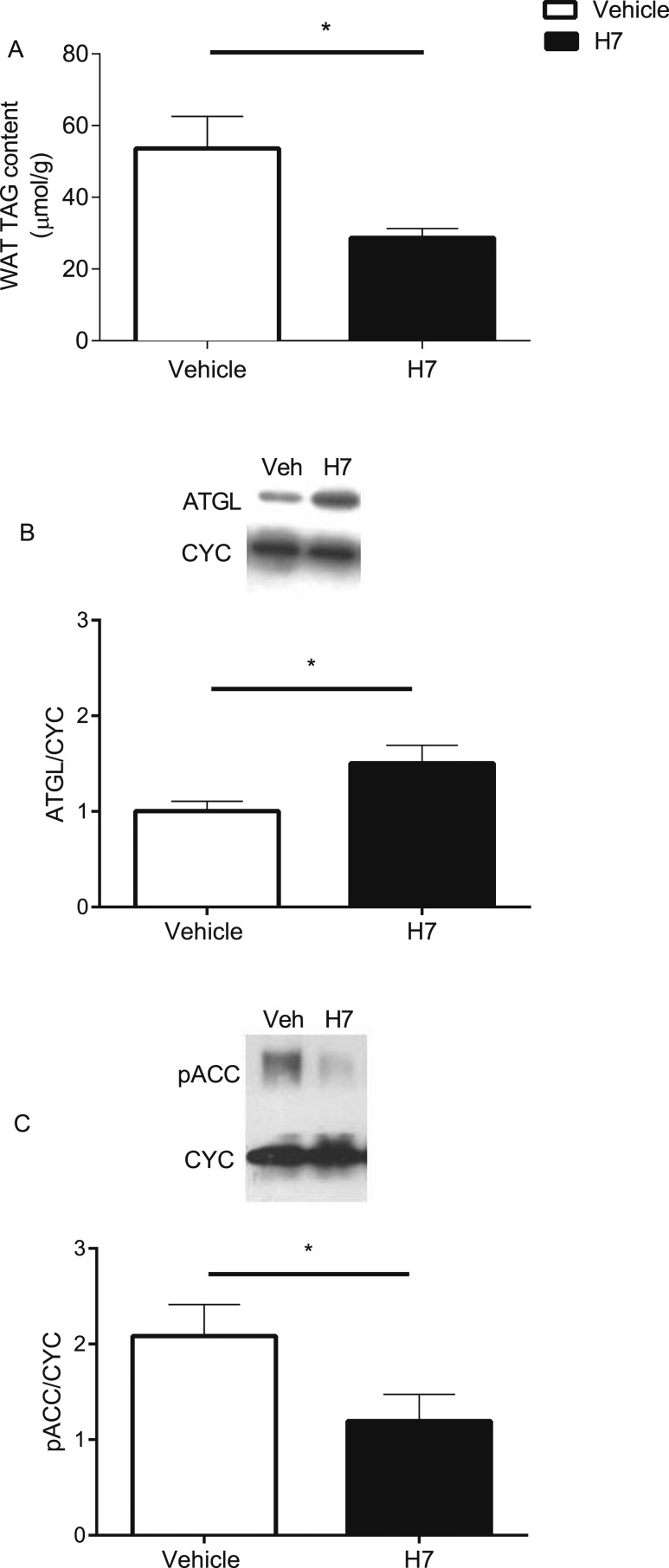
Effect of systemic treatment with a monoclonal antibody that targets the FGFR1c (H7) on WAT TG and protein content. (A) WAT TG content treated with vehicle or H7. (B) ATGL and (C) pACC protein content of WAT treated with vehicle or H7. Representative blots included. Values are group mean ± SEM (n = 8 per treatment). *P < 0.05 vs vehicle.

Hepatic (saline, 44.8 ± 1.7 µmol/g vs H7, 28.3 ± 1.4 µmol/g) and muscle (saline, 34.5 ± 1.1 µmol/g vs H7, 23.7 ± 1.8 µmol/g) TG content was also significantly reduced by 37% and 31%, respectively, (*P* < 0.05) in animals treated with H7. In WAT, TG content was significantly reduced (Fig. 4A; *P* < 0.05), ATGL protein content was significantly increased (Fig. 4B; *P* < 0.05), and pACC was significantly reduced (Fig. 4C; *P* < 0.05) in response to treatment with H7.

## Discussion

This study demonstrates that antibody-mediated targeting of FGFR1c exerts a potent glucose-lowering effect as a consequence of increased glucose uptake in white and brown adipose tissue. This effect is associated with increased basal cellular respiration, ATP generation, and nonmitochondrial respiration but not maximal oxygen consumption in adipocytes. However, there was no effect of treatment on whole-body energy expenditure, a possible consequence of contrasting changes in substrate utilization in other metabolically active tissues. Treatment with H7 was associated with increased markers of lipogenic (pACC) and lipolytic (ATGL) capacity in WAT, suggesting a futile (re)cycling of its TG content. Taken together, these data provide support for the notion that targeting this pathway in adipose tissue may have therapeutic potential for the treatment of metabolic syndrome (MetS).

It is now widely accepted that in addition to functioning as a daily buffer for circulating lipids, adipose tissue also plays a key role in the regulation of energy balance and glucose homeostasis via release of circulating metabolites and hormonal factors (11). Indeed, secreted adipokines have been investigated intensively for their roles in energy homeostasis (12) in an attempt to identify novel pathways for the treatment of obesity and its associated comorbidities. The endocrine FGFs have emerged as a novel family of hormonal factors that, when administered to rodents, have substantial antidiabetic activity (13). The downstream capabilities of FGF ligands are highly dependent upon engagement of FGFR1c, the predominant FGFR expressed in WAT and BAT (4). Indeed, mice with adipose-selective FGFR1 knockout were refractory to FGF21-induced improvements in glucose metabolism and body weight (8).

We previously reported that targeting FGFR1c in the Siberian hamster resulted in a profound antiobesogenic phenotype, with reduced blood glucose levels (5). Although this was supported by previous studies in mice (6), it was unknown which tissue(s) were responsible for this blood-glucose lowering effect and whether it was secondary to reduced food intake and body weight. The current study provides *in vivo* PET/CT data to indicate that H7-mediated lowering of blood glucose levels is predominantly associated with enhanced basal and insulin-stimulated glucose uptake in WAT and BAT. Although previous studies have shown that basal glucose uptake in WAT is low, under certain conditions (*e.g.*, stimulation with insulin and compounds such as *β*-agonists), this increases (14). In WAT, the H7-mediated effect observed in the current study is presumably a consequence of accelerated rates of TG hydrolysis (as indicated by the reduction in WAT weight and TG content) coupled to resynthesis, requiring large amounts of ATP (15). This notion of futile (re)cycling is supported by the increase in the protein content of ATGL (the rate-limiting step in TG hydrolysis) and reduction in pACC (indicating increased activity of a key enzyme in *de novo* lipogenesis) in WAT in response to treatment with H7, albeit favoring net hydrolysis (as indicated by the reduction in TG content). This futile cycling of TG requires a significant amount of ATP, and the increase in basal cellular respiration and ATP generation in 3T3-L1 adipocytes in response to H7 treatment supports this mechanism. Furthermore, the increase in glucose uptake *in vitro* demonstrates a direct effect of treatment rather than a consequence of reduced food intake and body weight *in vivo*.

Although the primary role of BAT is to maintain body temperature during the adaptive response to cold exposure, since its discovery in adult humans, it has emerged as an attractive target organ for the treatment of MetS. BAT displays a very high rate of glucose uptake, particularly under sympathetic activation (16). Furthermore, it is known to exert anti–type 2 diabetes mellitus (T2D) effects associated with improvements of dyslipidemia and insulin secretion, as well as decreased insulin resistance in T2D (17). In the current study, basal glucose uptake in BAT was 20% higher than in WAT in response to treatment. Furthermore, treatment was associated with reduced plasma insulin and TNF-*α*. Increasing BAT activity has advantageous effects on body composition and insulin sensitivity in mice, which suggests that BAT is an endocrine organ that can function to improve whole-body and tissue glucose homeostasis, effects mediated by TNF-*α*, interleukin-6, and the glucose transporter GLUT1, which is known to regulate insulin-independent glucose uptake (18, 19). It is likely that the redirection of glucose to energy combusting BAT without further storage in skeletal muscle (which showed a reduction in glucose uptake in response to treatment with H7) may contribute to the weight loss and insulin-sensitizing effect of H7. Overall, our data provides evidence that adipose tissue plays a dominant role in mediating the effects of targeting the FGFR1c, revealing the clinical potential of this pathway for the treatment of T2D and other chronic disorders linked to MetS and obesity.

Unlike adipose tissue, liver and skeletal muscle are not likely to be direct targets tissues for the H7 actions. Indeed, severe energy restriction (that is accompanied by weight loss of similar magnitude to the one observed in this study) leads to altered hepatic glucose uptake/metabolism and a shift in substrate utilization in skeletal muscle toward fat oxidation with a concomitant reduction in the oxidation of glucose and the development of insulin resistance in that tissue (20). Future investigations are also required to determine the precise role of FGFR1c-mediated glucose uptake in the brain and possible link to H7-mediated appetite regulation.

## Conclusion

The increasing prevalence of obesity and its comorbidities, including, but not limited to, T2D, are predicted to become two-thirds of the global burden of disease if current dietary and lifestyle trends continue (21). Existing therapies are unable to provide durable and robust change in metabolic health, and therefore it remains imperative to identify novel therapeutic agents able to effect meaningful change. The current study highlights the importance of adipose tissue FGFR1c as a target for an effective treatment strategy for MetS by reducing body weight, food intake, lipid accumulation, and thus lipotoxicity in adipose and nonadipose tissues, while lowering glycemia by enhancing glucose disposal primarily in adipose tissue (22–24).

## Appendix

**Appendix.** **Antibody Table**

**Peptide/Protein Target**	**Antigen Sequence (If Known)**	**Name of Antibody**	**Manufacturer, Catalog No.**	**Species Raised in; Monoclonal or Polyclonal**	**Dilution Used**	RRID
ATGL		Anti-Adipose Triglyceride Lipase antibody [EPR3444(2)]	Abcam, ab109251	Rabbit; monoclonal	1:1000	AB_10864772
pACC		Phospho-Acetyl-CoA Carboxylase (Ser79) antibody	New England Biolabs, 3661	Rabbit; polyclonal	1:1000	AB_330337
CYC		Anti-Cyclophilin B antibody (k2E2)	Abcam, ab74173	Mouse; monoclonal	1:4000	AB_1268387

Abbreviation: RRID, Research Resource Identifier.
